# Avelumab Maintenance Therapy in Patients With Curatively Unresectable Urothelial Carcinoma in Japan: Subgroup Analyses of Post‐Marketing Surveillance Data by Age, Prior Chemotherapy Regimen, and Best Overall Response to Prior Chemotherapy

**DOI:** 10.1002/cam4.71177

**Published:** 2025-10-30

**Authors:** Masayoshi Nagata, Eiji Kikuchi, Taito Ito, Masashi Sato, Mie Ogi, Makiko Morita, Masahiro Kajita, Hiroyuki Nishiyama

**Affiliations:** ^1^ Department of Urology Juntendo University Graduate School of Medicine Tokyo Japan; ^2^ Department of Urology St. Marianna University School of Medicine Kanagawa Japan; ^3^ Medical Department, Merck Biopharma Co., Ltd., Tokyo, Japan, an Affiliate of Merck KGaA Darmstadt Germany; ^4^ Research & Development, Merck Biopharma Co., Ltd., Tokyo, Japan, an Affiliate of Merck KGaA Darmstadt Germany; ^5^ Global Development Operations, Merck Biopharma Co., Ltd., Tokyo, Japan, an Affiliate of Merck KGaA Darmstadt Germany; ^6^ Global Patient Safety Japan, Merck Biopharma Co., Ltd., Tokyo, Japan, an Affiliate of Merck KGaA Darmstadt Germany; ^7^ Department of Urology, Faculty of Medicine University of Tsukuba Ibaraki Japan

**Keywords:** age, avelumab maintenance, platinum‐based chemotherapy, post‐marketing surveillance, real‐world data, urothelial carcinoma

## Abstract

**Introduction:**

Avelumab maintenance therapy was approved in Japan for curatively unresectable urothelial carcinoma (UC) without progression after prior platinum‐based chemotherapy (PBC) based on results from the JAVELIN Bladder 100 phase 3 trial. We report post hoc analyses of post‐marketing surveillance (PMS) data in subgroups defined by age, prior PBC regimen, and best overall response (BOR) to prior PBC.

**Methods:**

Patients with curatively unresectable UC who received ≥ 1 dose of avelumab maintenance in Japan between February and December 2021 were evaluated. The primary objective was to evaluate safety based on prespecified adverse drug reactions (ADRs). The secondary objective was to evaluate effectiveness, including time to treatment failure (TTF; discontinuation for any reason) and overall survival (OS).

**Results:**

The analysis population included 453 patients. In patients aged ≤ 64 (*n* = 75), 65 to 74 (*n* = 198), or ≥ 75 (*n* = 180) years, prespecified ADRs occurred in 17 (22.7%), 69 (34.9%), and 58 (32.2%); median TTF was 4.8, 4.4, and 4.9 months; and 12‐month OS rates were 77.6%, 82.7%, and 72.6%, respectively. In patients with prior cisplatin + gemcitabine (*n* = 267), carboplatin + gemcitabine (*n* = 163), or dose‐dense methotrexate, vinblastine, doxorubicin, and cisplatin (*n* = 9) treatment, prespecified ADRs occurred in 93 (34.8%), 45 (27.6%), and 3 (33.3%); median TTF was 4.6, 4.6, and 5.1 months; and 12‐month OS rates were 79.6%, 73.8%, and 88.9%, respectively. In patients with prior complete response (*n* = 47), partial response (*n* = 242), or stable disease (*n* = 149), prespecified ADRs occurred in 16 (34.0%), 79 (32.6%), and 45 (30.2%); median TTF was 5.2, 4.6, and 4.6 months; and 12‐month OS rates were 89.4%, 76.3%, and 75.8%, respectively.

**Discussion:**

In this PMS population, safety and effectiveness were observed with avelumab maintenance therapy across subgroups defined by age, prior PBC regimen, or BOR to prior PBC. Findings support the favorable benefit–risk profile of avelumab maintenance in clinical practice.

**Trial Registration:**

University Hospital Medical Information Network Clinical Trials Registry (UMIN‐CTR: UMIN 43435)

## 1 Introduction

Bladder cancer is the second most common cancer within the urinary system and the ninth most common cancer worldwide, and is associated with considerable morbidity and mortality [1]. In 2024 in Japan, bladder cancer was estimated to account for 24,700 new cases and 9900 attributable deaths [2]. Urothelial carcinoma (UC) accounts for approximately 90% of all bladder cancers, and UC may also arise in the upper urinary tract (renal pelvis or ureter) [3]. In Japan, incidences of upper‐ and lower‐tract UC are similar [4, 5, 6]. Despite several treatment developments, advanced UC (aUC; stage IV) is associated with poor prognosis [7].

Avelumab maintenance therapy is the recommended standard of care for cisplatin‐eligible or ‐ineligible patients with aUC who are progression‐free after receiving first‐line (1L) platinum‐based chemotherapy (PBC) in international treatment guidelines [8, 9, 10, 11]. The approval of avelumab maintenance therapy worldwide was based on results from the JAVELIN Bladder 100 phase 3 trial, which demonstrated significantly prolonged overall survival (OS) and progression‐free survival (PFS) with avelumab + best supportive care (BSC) versus BSC alone in patients with aUC without disease progression after 1L PBC [12, 13]. In long‐term analyses (≥ 2 years of follow‐up), median OS from randomization with avelumab + BSC versus BSC alone in the overall population was 23.8 versus 15.0 months (hazard ratio [HR], 0.76 [95% CI, 0.63–0.91]; *p* = 0.0036), and median PFS was 5.5 versus 2.1 months, respectively (HR, 0.54 [95% CI, 0.46–0.64]; *p* < 0.0001). The acceptable long‐term safety and tolerability profile of avelumab maintenance was also demonstrated [13]. In a post hoc subgroup analysis of patients enrolled in Japan (*n* = 73), efficacy and safety findings were generally consistent with those reported in the overall population of JAVELIN Bladder 100 [6].

In Japan, avelumab was approved in February 2021 as maintenance treatment for patients with curatively unresectable UC without disease progression after PBC, and it is recommended in Japanese Urological Association treatment guidelines [11, 14, 15]. Because JAVELIN Bladder 100 enrolled a limited number of patients in Japan (avelumab + BSC arm, *n* = 36) [6, 12], post‐marketing surveillance (PMS) was performed to evaluate the real‐world safety and effectiveness of avelumab maintenance in clinical practice in Japan (*N* = 453) [5]. Primary analyses of PMS data showed the safety and effectiveness of avelumab maintenance in the overall population. In the overall population of the PMS, prespecified adverse drug reactions (ADRs) of any grade occurred in 144 patients (31.8%); the most common prespecified ADRs were infusion reaction and thyroid dysfunction. Median time to treatment failure (TTF) was 4.6 months (95% CI, 3.8–5.3 months), and the 12‐month OS rate was 77.9% (95% CI, 73.7%–81.5%). Other real‐world studies in various countries have also demonstrated the effectiveness and safety of avelumab maintenance in clinical practice, including a small study in Japan (J‐AVENUE; *N* = 79) [4, 16, 17]. In J‐AVENUE, avelumab maintenance demonstrated a clinically meaningful benefit in patients with aUC 1 year after its approval in Japan [4].

Older age is a key risk factor for UC, which has a median age at diagnosis of 73 years [18]. However, older patients with aUC are less likely to receive systemic treatment [19, 20, 21, 22]. Because Japan has a rapidly aging population, including a higher proportion of individuals aged ≥ 65 years than in most other countries [23], real‐world data from older patients are highly relevant. In patients with aUC receiving PBC, the preferred chemotherapy regimen depends on whether patients are considered eligible to receive platinum. Platinum eligibility is generally defined by criteria including Eastern Cooperative Oncology Group performance status (ECOG PS), glomerular filtration rate, and comorbidities (such as hearing function, peripheral neuropathy, and cardiac function); age is not included in platinum eligibility criteria [9]. In platinum‐eligible patients, preferred PBC regimens are cisplatin + gemcitabine or (dose‐dense) methotrexate, vinblastine, doxorubicin, and cisplatin ([dd]MVAC), whereas in cisplatin‐ineligible patients, carboplatin + gemcitabine is preferred [8, 9].

In analyses from JAVELIN Bladder 100, OS and PFS were prolonged with avelumab + BSC versus BSC alone across a range of subgroups, including older patients (≥ 65 years, ≥ 75 years, or ≥ 80 years), patients whose 1L treatment was cisplatin + gemcitabine or carboplatin + gemcitabine, and patients whose best overall response (BOR) to 1L PBC was complete response (CR), partial response (PR), or stable disease (SD) [24, 25, 26]. However, limited data are available regarding real‐world outcomes with avelumab maintenance in different subgroups of patients in Japan.

Here, we report subgroup analyses of PMS data obtained from clinical practice in Japan in subgroups defined by age, prior PBC regimen, and BOR to prior PBC.

## 2 Material and Methods

### 2.1 Study Design and Patient Population

Full details of the design for PMS of avelumab maintenance in curatively unresectable UC in Japan have been reported previously [5]. In brief, this prospective, noninterventional PMS was conducted to evaluate safety and effectiveness in patients with curatively unresectable UC who received ≥ 1 dose of avelumab maintenance therapy following chemotherapy at multiple centers in Japan between February 24, 2021 (date of regulatory approval), and December 7, 2021. The observation period in all patients was ≤ 52 weeks from the first dose of avelumab. Data were collected via case report forms. This PMS was registered with the University Hospital Medical Information Network Clinical Trials Registry (UMIN‐CTR: UMIN 43435) and was conducted in accordance with the regulations of the Good Post‐marketing Study Practice (GPSP) Ministerial Ordinance in Japan. Approval from ethics committees and institutional review boards was obtained based on the requirements of each institution, and all patients provided written informed consent for publication [27].

In the current report, analyses of PMS data were performed in subgroups defined by age (≤ 64, 65–74, or ≥ 75 years), prior PBC received before avelumab (cisplatin + gemcitabine, carboplatin + gemcitabine, or [dd]MVAC), or BOR to prior PBC (CR, PR, or SD). BOR to prior PBC was determined by individual institutions, according to their standard practice.

### 2.2 Endpoints

The primary objective of the PMS was to evaluate the safety of avelumab maintenance therapy in clinical practice in Japan. Data for prespecified ADRs defined by the Japanese Risk Management Plan deemed to be associated with avelumab were collected [28]: adrenal insufficiency, colitis or severe diarrhea, encephalitis or meningitis, hepatic function disorders, infusion reaction, interstitial lung disease, myasthenia gravis, myocarditis, myositis or rhabdomyolysis, nerve disorders (including Guillain‐Barré syndrome), pancreatitis, pituitary disorders, renal disorders, thyroid dysfunction, and type 1 diabetes mellitus. In addition, occurrences of hematuria and urinary tract infection were collected. The secondary objective was to evaluate the effectiveness of avelumab maintenance therapy in clinical practice, including TTF (defined as time from the start of avelumab maintenance therapy to treatment discontinuation for any reason, including disease progression, ADR, patient preference, surgery, hospital transfer, or death) and OS (defined as time from the start of avelumab maintenance therapy to death from any cause).

### 2.3 Statistical Analyses

Safety data were aggregated by ADR type and severity and are presented as overall frequencies and percentages. Effectiveness data were estimated using the Kaplan–Meier method. Data for patient and disease characteristics, prior chemotherapy, and avelumab treatment were analyzed using descriptive statistics.

## 3 Results

### 3.1 Age Subgroups

Of 453 patients treated at 213 institutions by database lock on March 6, 2024, 75 (16.6%) were aged ≤ 64 years (median, 59 years), 198 (43.7%) were aged 65 to 74 years (median, 71 years), and 180 (39.7%) were aged ≥ 75 years (median, 79 years). Baseline characteristics were generally balanced across age subgroups (Table 1). However, the proportion of patients with an ECOG PS of 0 decreased with increasing age (82.7% vs. 71.7% vs. 67.2%), and higher proportions of older patients had renal impairment (29.3% vs. 36.4% vs. 40.0%) and had received carboplatin + gemcitabine as prior PBC (12.0% vs. 32.8% vs. 49.4%), respectively.

**TABLE 1:** Baseline characteristics at the start of avelumab maintenance in subgroups defined by age, prior PBC regimen, and BOR to prior PBC.

	Age group			Prior PBC regimen^a^			BOR to prior PBC^b^		
	≤ 64 years (*n* = 75)	65–74 years (*n* = 198)	≥ 75 years (*n* = 180)	GC (*n* = 267)	GCa (*n* = 163)	(dd)MVAC (*n* = 9)	CR (*n* = 47)	PR (*n* = 242)	SD (*n* = 149)
Age, median (range), years	59 (21–64)	71 (65–74)	79 (75–91)	72 (21–91)	75 (53–89)	73 (54–75)	72 (40–87)	73 (21–91)	73 (48–89)
≤ 64 years, *n* (%)	75 (100)	—	—	61 (22.8)	9 (5.5)	2 (22.2)	7 (14.9)	46 (19.0)	18 (12.1)
65–74 years, *n* (%)	—	198 (100)	—	120 (44.9)	65 (39.9)	6 (66.7)	23 (48.9)	102 (42.1)	67 (45.0)
≥ 75 years, *n* (%)	—	—	180 (100)	86 (32.2)	89 (54.6)	1 (11.1)	17 (36.2)	94 (38.8)	64 (43.0)
BMI, median (range), kg/m^2^	23.1 (9.8–33.9)	22.8 (15.2–33.2)	22.7 (13.4–31.7)	23.0 (9.8–33.9)	22.2 (14.4–32.0)	23.6 (18.7–28.9)	22.6 (13.4–30.1)	22.9 (9.8–33.9)	22.6 (15.9–32.3)
Sex, *n* (%)									
Male	55 (73.3)	151 (76.3)	127 (70.6)	203 (76.0)	113 (69.3)	8 (88.9)	36 (76.6)	177 (73.1)	106 (71.1)
Female	20 (26.7)	47 (23.7)	53 (29.4)	64 (24.0)	50 (30.7)	1 (11.1)	11 (23.4)	65 (26.9)	43 (28.9)
ECOG PS, *n* (%)									
0	62 (82.7)	142 (71.7)	121 (67.2)	201 (75.3)	111 (68.1)	2 (22.2)	37 (78.7)	182 (75.2)	98 (65.8)
1	11 (14.7)	51 (25.8)	49 (27.2)	56 (21.0)	45 (27.6)	7 (77.8)	8 (17.0)	53 (21.9)	43 (28.9)
≥ 2	2 (2.6)	5 (2.5)	10 (5.5)	10 (3.7)	7 (4.3)	0	2 (4.3)	7 (2.9)	8 (5.3)
Primary tumor site, *n* (%)									
Bladder	42 (56.0)	113 (57.1)	89 (49.4)	152 (56.9)	77 (47.2)	7 (77.8)	28 (59.6)	124 (51.2)	81 (54.4)
Upper tract	33 (44.0)	85 (42.9)	91 (50.6)	115 (43.1)	86 (52.8)	2 (22.2)	19 (40.4)	118 (48.8)	68 (45.6)
Metastatic lesion, *n* (%)									
Absent	6 (8.0)	15 (7.6)	23 (12.8)	21 (7.9)	23 (14.1)	0	11 (23.4)	11 (4.5)	20 (13.4)
Present^c^	69 (92.0)	183 (92.4)	157 (87.2)	246 (92.1)	140 (85.9)	9 (100.0)	36 (76.6)	231 (95.5)	129 (86.6)
Lung	18 (26.1)	44 (24.0)	47 (29.9)	63 (25.6)	40 (28.6)	2 (22.2)	10 (27.8)	57 (24.7)	40 (31.0)
Bone	9 (13.0)	26 (14.2)	20 (12.7)	40 (16.3)	11 (7.9)	2 (22.2)	4 (11.1)	29 (12.6)	19 (14.7)
Liver	10 (14.5)	15 (8.2)	14 (8.9)	27 (11.0)	12 (8.6)	0	6 (16.7)	20 (8.7)	13 (10.1)
Lymph node	56 (81.2)	139 (76.0)	114 (72.6)	187 (76.0)	107 (76.4)	7 (77.8)	28 (77.8)	181 (78.4)	89 (69.0)
Other	7 (10.1)	24 (13.0)	20 (12.8)	27 (11.0)	18 (12.9)	2 (22.2)	7 (19.5)	30 (13.0)	14 (10.9)
Disease stage, *n* (%)									
Stage III	4 (5.3)	10 (5.1)	14 (7.8)	11 (4.1)	17 (10.4)	0	3 (4.1)	7 (2.9)	17 (11.4)
Stage IV	66 (88.0)	180 (90.9)	158 (87.8)	240 (89.9)	142 (87.1)	9 (100.0)	37 (78.7)	226 (93.4)	128 (85.9)
Other	5 (6.7)	8 (4.0)	8 (4.4)	16 (4.0)	4 (2.5)	0	7 (14.9)	9 (3.7)	4 (2.7)
Comorbidity, *n* (%)^c^									
Renal impairment	22 (29.3)	72 (36.4)	72 (40.0)	77 (28.8)	82 (50.3)	2 (22.2)	11 (23.4)	93 (38.4)	57 (38.3)
Hepatic impairment	3 (4.0)	4 (2.0)	5 (2.8)	7 (2.6)	5 (3.1)	0	2 (4.3)	5 (2.1)	4 (2.7)
Interstitial lung disease	1 (1.3)	1 (0.5)	4 (2.2)	4 (1.5)	2 (1.2)	0	1 (2.1)	3 (1.2)	2 (1.3)
Autoimmune disease	0	1 (0.5)	3 (1.7)	0	4 (2.5)	0	1 (2.1)	2 (0.8)	1 (0.7)
Other	25 (33.3)	74 (37.4)	89 (49.4)	108 (40.4)	66 (40.5)	5 (55.6)	16 (34.0)	105 (43.4)	61 (40.9)
Prior treatment, *n* (%)^c^									
Surgery	52 (69.3)	151 (76.3)	130 (72.2)	196 (73.4)	120 (73.6)	5 (55.6)	39 (83.0)	174 (71.9)	111 (74.5)
Radiation therapy	8 (10.7)	28 (14.1)	27 (15.0)	35 (13.1)	22 (13.5)	3 (33.3)	5 (10.6)	36 (14.9)	17 (11.4)
Prior PBC regimen, *n* (%)									
GC	61 (81.3)	120 (60.6)	86 (47.8)	267 (100)	—	—	36 (76.6)	154 (63.6)	66 (44.3)
GCa	9 (12.0)	65 (32.8)	89 (49.4)	—	163 (100)	—	11 (23.4)	79 (32.6)	71 (47.7)
(dd)MVAC	2 (2.7)	6 (3.0)	1 (0.6)	—	—	9 (100)	0	5 (2.1)	3 (2.0)
Unknown	3 (4.0)	7 (3.5)	4 (2.2)	—	—	—	0	4 (1.7)	9 (6.0)
BOR to prior PBC, *n* (%)									
CR	7 (9.3)	23 (11.6)	17 (9.4)	36 (13.5)	11 (7.6)	0	47 (100)	—	—
PR	46 (61.3)	102 (51.5)	94 (52.2)	154 (57.7)	79 (48.5)	5 (55.6)	—	242 (100)	—
SD	18 (24.0)	67 (33.8)	64 (35.6)	66 (24.7)	71 (43.6)	3 (33.3)	—	—	149 (100)
Unknown	4 (5.3)	6 (3.0)	5 (2.8)	11 (4.2)	2 (1.2)	1 (11.1)	—	—	—
Treatment‐free interval, *n* (%)									
< 4 weeks	32 (42.7)	88 (44.4)	84 (46.7)	118 (44.2)	79 (48.5)	6 (66.7)	20 (42.6)	117 (48.3)	61 (40.9)
4 to < 6 weeks	13 (17.3)	45 (22.7)	38 (21.1)	53 (19.9)	38 (23.3)	0	12 (25.5)	44 (18.2)	37 (24.8)
6 to < 8 weeks	13 (17.3)	27 (13.6)	27 (15.0)	42 (15.7)	23 (14.1)	1 (11.1)	3 (6.4)	41 (16.9)	22 (14.8)
8 to 10 weeks	5 (6.7)	14 (7.1)	7 (3.9)	16 (6.0)	7 (4.3)	0	1 (2.1)	13 (5.4)	11 (7.4)
> 10 weeks	12 (16.0)	24 (12.1)	24 (13.3)	38 (14.2)	16 (9.8)	2 (22.2)	11 (23.4)	27 (11.2)	18 (12.1)

Abbreviations: (dd)MVAC, (dose‐dense) methotrexate, vinblastine, doxorubicin, and cisplatin; BMI, body mass index; BOR, best overall response; CR, complete response; ECOG PS, Eastern Cooperative Oncology Group performance status; GC, gemcitabine + cisplatin; GCa, gemcitabine + carboplatin; PBC, platinum‐based chemotherapy; PR, partial response; SD, stable disease.

^**a**^ Prior PBC regimen was unknown in 14 patients.

^b^ BOR to prior PBC was unknown in 15 patients.

^c^ Patients may be included in ≥ 1 category.

In patients aged ≤ 64, 65 to 74, or ≥ 75 years, prespecified ADRs of any grade occurred in 17 (22.7%), 69 (34.9%), and 58 (32.2%), including grade ≥ 3 events in 2 (2.7%), 15 (7.6%), and 18 (10.0%), respectively (Table 2). Frequencies of individual ADRs were generally similar across age subgroups (Table 3). In patients aged ≤ 64, 65 to 74, or ≥ 75 years, corticosteroid treatment of any dose was administered for ADRs in 3 (4.0%), 19 (9.6%), and 16 (8.9%), and high‐dose corticosteroid treatment (≥ 40 mg daily dose of prednisone or equivalent) was administered in 2 (2.7%), 10 (5.1%), and 7 (3.9%), respectively (Table 2).

**TABLE 2:** Summary of prespecified ADRs and corticosteroid treatment for prespecified ADRs.

	Age group			Prior PBC regimen^a^			BOR to prior PBC^b^		
	≤ 64 years (*n* = 75)	65–74 years (*n* = 198)	≥ 75 years (*n* = 180)	GC (*n* = 267)	GCa (*n* = 163)	(dd)MVAC (*n* = 9)	CR (*n* = 47)	PR (*n* = 242)	SD (*n* = 149)
Prespecified ADR, *n* (%)									
Any grade	17 (22.7)	69 (34.9)	58 (32.2)	93 (34.8)	45 (27.6)	3 (33.3)	16 (34.0)	79 (32.6)	45 (30.2)
Grade ≥ 3	2 (2.7)	15 (7.6)	18 (10.0)	22 (8.2)	12 (7.4)	0	4 (8.5)	16 (6.6)	13 (8.7)
Leading to death	0	0	1 (0.6)	1 (0.4)	0	0	0	1 (0.4)	0
Corticosteroid treatment, *n* (%)									
Any dose	3 (4.0)	19 (9.6)	16 (8.9)	23 (8.6)	14 (8.6)	0	4 (8.5)	20 (8.3)	14 (9.4)
High dose^c^	2 (2.7)	10 (5.1)	7 (3.9)	10 (3.7)	8 (4.9)	0	2 (4.3)	11 (4.5)	6 (4.0)

Abbreviations: (dd)MVAC, (dose‐dense) methotrexate, vinblastine, doxorubicin, and cisplatin; ADR, adverse drug reaction; BOR, best overall response; CR, complete response; GC, gemcitabine + cisplatin; GCa, gemcitabine + carboplatin; PBC, platinum‐based chemotherapy; PR, partial response; SD, stable disease.

^**a**^ Prior PBC regimen was unknown in 14 patients.

^b^ BOR to prior PBC was unknown in 15 patients.

^c^ ≥ 40 mg daily dose of prednisone or equivalent.

**TABLE 3:** Prespecified ADRs in subgroups defined by age, prior PBC, and BOR to prior PBC.

Age subgroups									
	≤ 64 years (*n* = 75)			65–74 years (*n* = 198)			≥ 75 years (*n* = 180)		
	Grade 1/2	Grade 3/4	Grade 5	Grade 1/2	Grade 3/4	Grade 5	Grade 1/2	Grade 3/4	Grade 5
Infusion reaction	6 (8.0)	0	0	22 (11.1)	1 (0.5)	0	22 (12.2)	2 (1.1)	0
Thyroid dysfunction	3 (4.0)	1 (1.3)	0	20 (10.1)	0	0	9 (5.0)	0	0
Interstitial lung disease	0	0	0	6 (3.0)	2 (1.0)	0	5 (2.8)	3 (1.7)	0
Hepatic function disorders	5 (6.7)	0	0	3 (1.5)	2 (1.0)	0	4 (2.2)	0	0
Nerve disorders	1 (1.3)	0	0	3 (1.5)	2 (1.0)	0	2 (1.1)	2 (1.1)	1 (0.6)
Myositis or rhabdomyolysis	1 (1.3)	1 (1.3)	0	2 (1.0)	2 (1.0)	0	1 (0.6)	1 (0.6)	0
Colitis or severe diarrhea	0	0	0	2 (1.0)	2 (1.0)	0	1 (0.6)	2 (1.1)	0
Adrenal insufficiency	0	0	0	1 (0.5)	3 (1.5)	0	0	3 (1.7)	0
Renal disorders	0	0	0	1 (0.5)	1 (0.5)	0	2 (1.1)	2 (1.1)	0
Pituitary disorders	0	0	0	0	2 (1.0)	0	0	0	0
Type 1 diabetes mellitus	0	0	0	0	2 (1.0)	0	0	0	0
Encephalitis or meningitis	0	0	0	0	1 (0.5)	0	0	0	1 (0.6)
Pancreatitis	0	0	0	0	0	0	0	1 (0.6)	0
Myasthenia gravis	0	0	0	0	0	0	0	0	0
Myocarditis	0	0	0	0	0	0	0	0	0
Hematuria	1 (1.3)	0	0	1 (0.5)	0	0	1 (0.6)	2 (1.1)	0
Urinary tract infection	1 (1.3)	0	0	0	0	0	1 (0.6)	0	0

Subgroups defined by prior PBC^a^									
	GC (*n* = 267)			GCa (*n* = 163)			(dd)MVAC (*n* = 9)		
	Grade 1/2	Grade 3/4	Grade 5	Grade 1/2	Grade 3/4	Grade 5	Grade 1/2	Grade 3/4	Grade 5
Infusion reaction	33 (12.4)	2 (0.8)	0	15 (9.2)	1 (0.6)	0	2 (22.2)	0	0
Thyroid dysfunction	20 (7.5)	1 (0.4)	0	10 (6.1)	0	0	1 (11.1)	0	0
Interstitial lung disease	7 (2.6)	2 (0.8)	0	4 (2.5)	3 (1.8)	0	0	0	0
Hepatic function disorders	10 (3.8)	2 (0.8)	0	1 (0.6)	0	0	0	0	0
Nerve disorders	4 (1.5)	4 (1.5)	1 (0.4)	2 (1.2)	0	0	0	0	0
Myositis or rhabdomyolysis	4 (1.5)	4 (1.5)	0	0	0	0	0	0	0
Colitis or severe diarrhea	1 (0.4)	0	0	1 (0.6)	3 (1.8)	0	0	0	0
Adrenal insufficiency	1 (0.4)	5 (1.9)	0	0	1 (0.6)	0	0	0	0
Renal disorders	0	0	0	3 (1.8)	3 (1.8)	0	0	0	0
Pituitary disorders	0	2 (0.8)	0	0	0	0	0	0	0
Type 1 diabetes mellitus	0	1 (0.4)	0	0	1 (0.6)	0	0	0	0
Encephalitis or meningitis	0	1 (0.4)	1 (0.4)	0	0	0	0	0	0
Pancreatitis	0	0	0	0	1 (0.6)	0	0	0	0
Myasthenia gravis	0	0	0	0	0	0	0	0	0
Myocarditis	0	0	0	0	0	0	0	0	0
Hematuria	2 (0.8)	1 (0.4)	0	1 (0.6)	1 (0.6)	0	0	0	0
Urinary tract infection	1 (0.4)	0	0	1 (0.6)	0	0	0	0	0

Subgroups defined by BOR prior PBC^b^									
	CR (*n* = 47)			PR (*n* = 242)			SD (*n* = 149)		
	Grade 1/2	Grade 3/4	Grade 5	Grade 1/2	Grade 3/4	Grade 5	Grade 1/2	Grade 3/4	Grade 5
Infusion reaction	5 (10.6)	0	0	30 (12.4)	0	0	15 (10.1)	3 (2.0)	0
Thyroid dysfunction	4 (8.5)	1 (2.1)	0	19 (7.9)	0	0	9 (6.0)	0	0
Interstitial lung disease	2 (4.3)	0	0	8 (3.3)	2 (0.8)	0	1 (0.7)	3 (2.0)	0
Hepatic function disorders	0	0	0	7 (2.9)	1 (0.4)	0	2 (1.3)	1 (0.7)	0
Nerve disorders	2 (4.3)	2 (4.3)	0	4 (1.7)	2 (0.8)	1 (0.4)	0	0	0
Myositis or rhabdomyolysis	1 (2.1)	0	0	2 (0.8)	1 (0.4)	0	1 (0.7)	2 (1.3)	0
Colitis or severe diarrhea	0	0	0	3 (1.2)	1 (0.4)	0	0	3 (2.0)	0
Adrenal insufficiency	0	0	0	0	6 (2.5)	0	1 (0.7)	0	0
Renal disorders	0	1 (2.1)	0	2 (0.8)	1 (0.4)	0	1 (0.7)	1 (0.7)	0
Pituitary disorders	0	0	0	0	2 (0.8)	0	0	0	0
Type 1 diabetes mellitus	0	0	0	0	1 (0.4)	0	0	1 (0.7)	0
Encephalitis or meningitis	0	0	0	0	1 (0.4)	1 (0.4)	0	0	0
Pancreatitis	0	0	0	0	0	0	0	1 (0.7)	0
Myasthenia gravis	0	0	0	0	0	0	0	0	0
Myocarditis	0	0	0	0	0	0	0	0	0
Hematuria	0	0	0	1 (0.4)	1 (0.4)	0	2 (1.3)	0	0
Urinary tract infection	0	0	0	1 (0.4)	0	0	1 (0.7)	0	0

*Note:* Infusion reaction includes infusion‐related reaction, chills, and pyrexia. One grade 5 ADR of noninfective encephalitis was observed in 1 patient, which was categorized as a prespecified ADR for both nerve disorder and encephalitis.

Abbreviations: (dd)MVAC, (dose‐dense) methotrexate, vinblastine, doxorubicin, and cisplatin; ADR, adverse drug reaction, BOR, best overall response; CR, complete response; GC, gemcitabine + cisplatin; GCa, gemcitabine + carboplatin; PBC, platinum‐based chemotherapy; PR, partial response; SD, stable disease.

^**a**^ Prior PBC regimen was unknown in 14 patients.

^b^ BOR to prior PBC was unknown in 15 patients.

**FIGURE 1:**
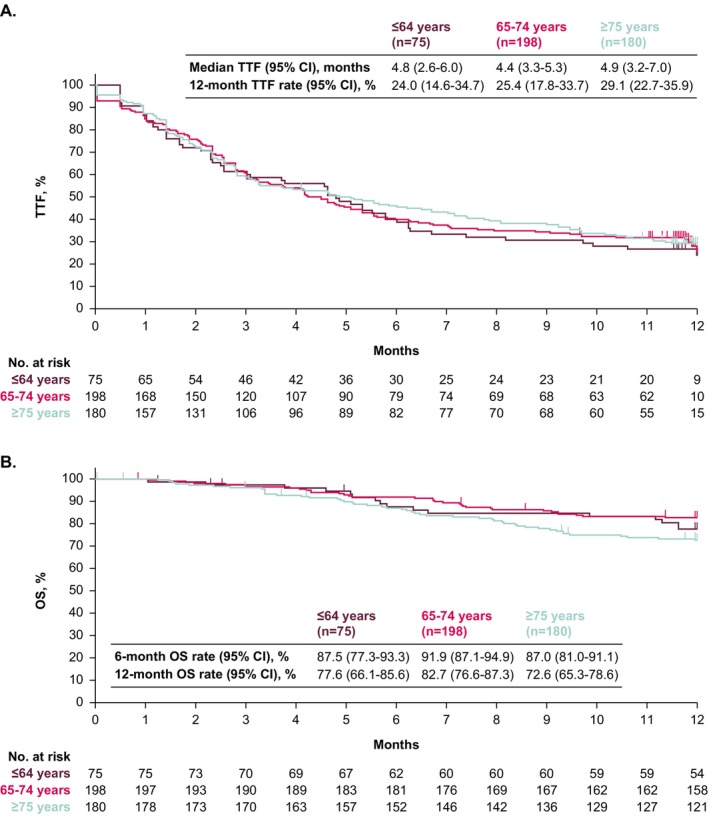
Kaplan–Meier analysis of (A) TTF and (B) OS in subgroups defined by age. OS, overall response; TTF, time to treatment failure.

In patients aged ≤ 64, 65 to 74, or ≥ 75 years, median TTF from the start of avelumab maintenance was 4.8 months (95% CI, 2.6–6.0), 4.4 months (95% CI, 3.3–5.3), and 4.9 months (95% CI, 3.2–7.0); 12‐month TTF rates were 24.0% (95% CI, 14.6–34.7), 25.4% (95% CI, 17.8–33.7), and 29.1% (95% CI, 22.7–35.9), respectively (Figure 1A). Median OS was not reached in any subgroup, and 12‐month OS rates were 77.6% (95% CI, 66.1–85.6), 82.7% (95% CI, 76.6%–87.3%), and 72.6% (95% CI, 65.3%–78.6%), respectively (Figure 1B).

At the end of the observation period, avelumab maintenance therapy was ongoing in 19 patients (25.3%) aged ≤ 64 years, 59 patients (29.8%) aged 65 to 74 years, and 53 patients (29.4%) aged ≥ 75 years (Table 4). In all age subgroups, the most common reason for treatment discontinuation was disease progression in 64.6% to 76.8% of patients. In patients who discontinued avelumab and received next‐line anticancer drug treatment, the most common regimens in patients aged ≤ 64 years and 65 to 74 years were cisplatin + gemcitabine (37.5% and 33.3%, respectively) and enfortumab vedotin (32.5% and 23.5%, respectively), whereas in patients aged ≥ 75 years, carboplatin + gemcitabine (28.6%) and pembrolizumab (27.0%) were the most common next‐line regimens (Table 4).

**TABLE 4:** Treatment disposition at the end of the observation period and subsequent treatment.

	Age group			Prior PBC regimen^a^			BOR to prior PBC^b^		
	≤ 64 years (*n* = 75)	65–74 years (*n* = 198)	≥ 75 years (*n* = 180)	GC (*n* = 267)	GCa (*n* = 163)	(dd)MVAC (*n* = 9)	CR (*n* = 47)	PR (*n* = 242)	SD (*n* = 149)
Avelumab maintenance therapy ongoing, *n* (%)	19 (25.3)	59 (29.8)	53 (29.4)	74 (27.7)	51 (31.3)	2 (22.2)	17 (36.2)	67 (27.7)	42 (28.2)
Avelumab discontinued, *n* (%)	56 (74.7)	139 (70.2)	127 (70.6)	193 (72.3)	112 (68.7)	7 (77.8)	30 (63.8)	175 (72.3)	107 (71.8)
Reason for discontinuation, *n* (%)^c^	***n* = 56**	***n* = 139**	***n* = 127**	***n* = 193**	***n* = 112**	***n* = 7**	***n* = 30**	***n* = 175**	***n* = 107**
Disease progression	43 (76.8)	98 (70.5)	82 (64.6)	130 (67.4)	79 (70.5)	5 (71.4)	18 (60.0)	118 (67.4)	79 (73.8)
Prespecified ADRs	3 (5.4)	16 (11.5)	13 (10.2)	23 (11.9)	9 (8.0)	0	4 (13.3)	19 (10.9)	9 (8.4)
Surgery	2 (3.6)	3 (2.2)	2 (1.6)	5 (2.6)	2 (1.8)	0	0	6 (3.4)	1 (0.9)
Patient preference	2 (3.6)	8 (5.8)	15 (11.8)	17 (8.8)	6 (5.4)	1 (14.3)	3 (10.0)	12 (6.9)	9 (8.4)
Death	1 (1.8)	0	3 (2.4)	2 (1.0)	2 (1.8)	0	0	4 (2.3)	0
Transfer to another hospital	2 (3.6)	1 (0.7)	2 (1.6)	1 (0.5)	3 (2.7)	0	0	3 (1.7)	2 (1.9)
Other	4 (7.1)	15 (10.8)	12 (9.4)	16 (8.3)	13 (11.6)	1 (14.3)	5 (16.7)	13 (7.4)	12 (11.2)
Received subsequent treatment, *n*/*N* (%)^d^	40/56 (71.4)	81/139 (58.3)	63/127 (49.6)	115/193 (59.6)	59/112 (52.7)	5/7 (71.4)	15/30 (50.0)	103/175 (58.9)	57/107 (53.3)
Type of subsequent treatment received, *n* (%)^e^	***n* = 40**	***n* = 81**	***n* = 63**	***n* = 115**	***n* = 59**	***n* = 5**	***n* = 15**	***n* = 103**	***n* = 57**
Any chemotherapy	23 (57.5)	52 (64.2)	34 (54.0)	69 (60.0)	35 (59.3)	3 (60.0)	9 (60.0)	65 (63.1)	32 (56.1)
GC	15 (37.5)	27 (33.3)	11 (17.5)	50 (43.5)	2 (3.4)	1 (20.0)	7 (46.7)	37 (35.9)	7 (12.3)
GCa	5 (12.5)	11 (13.6)	18 (28.6)	6 (5.2)	27 (45.8)	1 (20.0)	1 (6.7)	18 (17.5)	15 (26.3)
Other combination	1 (2.5)	10 (12.3)	3 (4.8)	8 (7.0)	3 (5.1)	1 (20.0)	1 (6.7)	5 (4.9)	7 (12.3)
Monotherapy	2 (5.0)	4 (4.9)	2 (3.2)	5 (4.3)	3 (5.1)	0	0	5 (4.9)	3 (5.3)
Enfortumab vedotin	13 (32.5)	19 (23.5)	12 (19.0)	26 (22.6)	13 (22.0)	2 (40.0)	4 (26.7)	22 (21.4)	15 (26.3)
Pembrolizumab	4 (10.0)	9 (11.1)	17 (27.0)	20 (17.4)	10 (16.9)	0	1 (6.7)	16 (15.5)	10 (17.5)
Other	0	1 (1.2)	0	0	1 (1.7)	0	1 (6.7)	0	0

Abbreviations: (dd)MVAC, (dose‐dense) methotrexate, vinblastine, doxorubicin, and cisplatin; ADR, adverse drug reaction; BOR, best overall response; CR, complete response; GC, gemcitabine + cisplatin; GCa, gemcitabine + carboplatin; PBC, platinum‐based chemotherapy; PR, partial response; SD, stable disease.

^**a**^ Prior PBC regimen was unknown in 14 patients.

^**b**^ BOR to prior PBC was unknown in 15 patients.

^c^ Some patients were included in ≥ 1 category.

^d^ Percentages calculated using the total number of patients who discontinued avelumab maintenance treatment as the denominator.

^e^ Percentages calculated using the total number of patients who received subsequent treatment.

### 3.2 Subgroups Defined by Prior PBC

Of 453 patients enrolled, prior PBC regimen was cisplatin + gemcitabine in 267 (58.9%), carboplatin + gemcitabine in 163 (36.0%), and (dd)MVAC in 9 (2.0%); in 14 patients, the prior PBC regimen was unknown. Baseline characteristics were generally balanced across PBC subgroups (Table 1); however, compared with the cisplatin + gemcitabine and (dd)MVAC subgroups, the carboplatin + gemcitabine subgroup included a lower proportion of patients aged ≤ 64 years (22.8% and 22.2% vs. 5.5%), a higher proportion aged ≥ 75 years (32.2% and 11.1% vs. 54.6%), and a higher proportion with renal impairment (28.8% and 22.2% vs. 50.3%).

In the cisplatin + gemcitabine, carboplatin + gemcitabine, and (dd)MVAC subgroups, prespecified ADRs of any grade occurred in 93 (34.8%), 45 (27.6%), and 3 (33.3%), including grade ≥ 3 events in 22 (8.2%), 12 (7.4%), and 0, respectively (Table 2). The frequency of individual prespecified ADRs was generally similar between the cisplatin + gemcitabine and carboplatin + gemcitabine subgroups; the small number of patients in the (dd)MVAC subgroup hindered comparisons (Table 3). In patients treated with cisplatin + gemcitabine or carboplatin + gemcitabine, any‐dose corticosteroid treatment for ADRs was administered to 23 (8.6%) and 14 (8.6%), with high‐dose corticosteroid treatment in 10 (3.7%) and 8 (4.9%), respectively (Table 2). No patient in the (dd)MVAC subgroup received corticosteroid treatment for ADRs.

**FIGURE 2:**
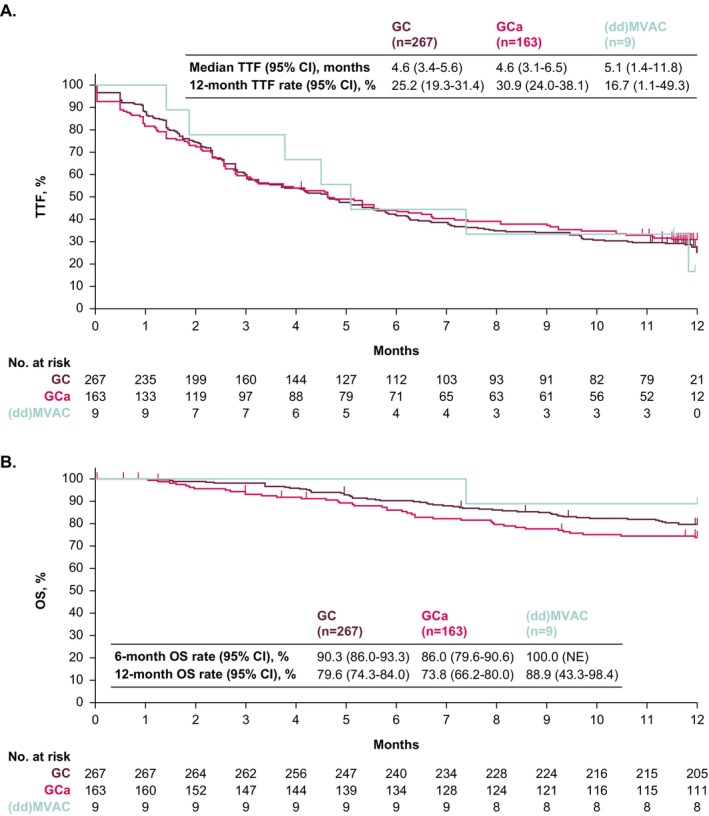
Kaplan–Meier analysis of (A) TTF and (B) OS in subgroups defined by prior PBC regimen. Prior PBC regimen was unknown in 14 patients. (dd)MVAC, (dose‐dense) methotrexate, vinblastine, doxorubicin, and cisplatin; GC, gemcitabine + cisplatin; GCa, gemcitabine + carboplatin; OS, overall survival; PBC, platinum‐based chemotherapy; TTF, time to treatment failure.

In patients treated with cisplatin + gemcitabine, carboplatin + gemcitabine, or (dd)MVAC, median TTF from the start of avelumab maintenance was 4.6 months (95% CI, 3.4–5.6), 4.6 months (95% CI, 3.1–6.5), and 5.1 months (95% CI, 1.4–11.8); 12‐month TTF rates were 25.2% (95% CI, 19.3–31.4), 30.9% (95% CI, 24.0–38.1), and 16.7% (95% CI, 1.1–49.3), respectively (Figure 2A). Median OS was not reached in any subgroup, and 12‐month OS rates were 79.6% (95% CI, 74.3–84.0), 73.8% (95% CI, 66.2–80.0), and 88.9% (95% CI, 43.3–98.4), respectively (Figure 2B).

At the end of the observation period, avelumab maintenance therapy was ongoing in 74 patients (27.7%) in the cisplatin + gemcitabine subgroup, 51 patients (31.3%) in the carboplatin + gemcitabine subgroup, and 2 patients (22.2%) in the (dd)MVAC subgroup (Table 4). In all subgroups, the most common reason for treatment discontinuation was disease progression in 67.4% to 71.4% of patients. In patients who discontinued avelumab and received next‐line anticancer drug treatment, the most common subsequent regimens in the cisplatin + gemcitabine subgroup were cisplatin + gemcitabine (43.5%) and enfortumab vedotin (22.6%), in the carboplatin + gemcitabine subgroup were carboplatin + gemcitabine (45.8%) and enfortumab vedotin (22.0%), and in the (dd)MVAC subgroup was enfortumab vedotin (40.0%) (Table 4).

### 3.3 Subgroups Defined by BOR to Prior PBC

Of 453 patients enrolled, BOR to prior PBC was CR in 47 (10.4%), PR in 242 (53.4%), and SD in 149 (32.9%); in 15 patients, BOR to prior PBC was unknown. Baseline characteristics were generally balanced across BOR subgroups (Table 1); however, compared with the CR subgroup, the PR and SD subgroups included a higher proportion of patients with renal impairment (23.4% vs. 38.4% and 38.3%) and with prior carboplatin + gemcitabine treatment (23.4% vs. 32.6% and 47.7%).

In the prior CR, PR, and SD subgroups, prespecified ADRs of any grade occurred in 16 (34.0%), 79 (32.6%), and 45 (30.2%), including grade ≥ 3 events in 4 (8.5%), 17 (7.0%), and 13 (8.7%), respectively (Table 2). Frequencies of individual ADRs were generally similar across prior BOR subgroups (Table 3). In the prior CR, PR, and SD subgroups, any‐dose corticosteroid treatment for ADRs was administered to 4 (8.5%), 20 (8.3%), and 14 (9.4%); high‐dose corticosteroid treatment was administered to 2 (4.3%), 11 (4.5%), and 6 (4.0%), respectively (Table 2).

**FIGURE 3:**
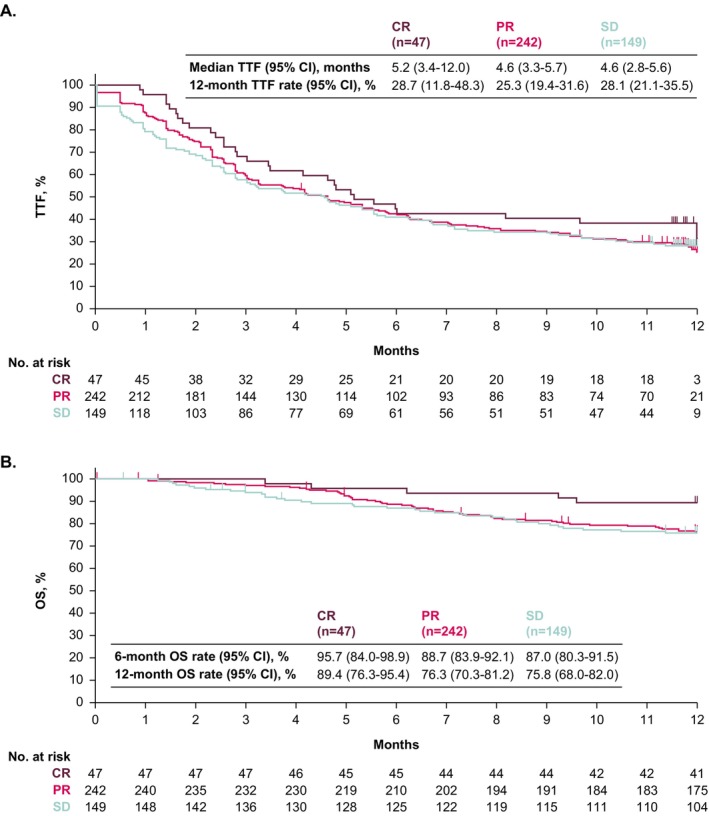
Kaplan–Meier analysis of (A) TTF and (B) OS in subgroups defined by BOR to prior PBC. BOR to prior PBC was unknown in 15 patients. BOR, best overall response; CR, complete response; OS, overall survival; PBC, platinum‐based chemotherapy; PR, partial response; SD, stable disease; TTF, time to treatment failure.

In the prior CR, PR, and SD subgroups, median TTF from the start of avelumab maintenance was 5.2 months (95% CI, 3.4–12.0), 4.6 months (95% CI, 3.3–5.7), and 4.6 months (95% CI, 2.8–5.6); 12‐month TTF rates were 28.7% (95% CI, 11.8–48.3), 25.3% (95% CI, 19.4–31.6), and 28.1% (95% CI, 21.1–35.5) (Figure 3A). Median OS was not reached in any subgroup, and 12‐month OS rates were 89.4% (95% CI, 76.3–95.4), 76.3% (95% CI, 70.3–81.2), and 75.8% (95% CI, 68.0–82.0), respectively (Figure 3B).

At the end of the observation period, avelumab maintenance therapy was ongoing in 17 patients (36.2%) with prior CR, 67 patients (27.7%) with prior PR, and 42 patients (28.2%) with prior SD (Table 4). In all subgroups, the most common reason for avelumab discontinuation was disease progression in 60.0% to 73.8% of patients. In patients who discontinued avelumab and received next‐line anticancer drug treatment, the most common regimens in the CR and PR subgroups were cisplatin + gemcitabine (46.7% and 35.9%, respectively) and enfortumab vedotin (26.7% and 21.4%, respectively), and in the SD subgroup were carboplatin + gemcitabine and enfortumab vedotin (both 26.3%) (Table 4).

## 4 Discussion

This PMS population provides a large and comprehensive dataset of patients with curatively unresectable UC who received avelumab maintenance therapy in clinical practice in Japan. Final analyses from the overall PMS population demonstrated the safety and effectiveness of avelumab maintenance in clinical practice, and outcomes were broadly consistent with findings from the JAVELIN Bladder 100 phase 3 trial and real‐world studies in other countries [5, 6, 12, 13, 16, 17]. Subgroup analyses reported here suggest that the safety and effectiveness of avelumab maintenance therapy were generally similar across subgroups defined by patient age, prior PBC regimen received, or BOR to prior PBC, which was also reported in analyses from the JAVELIN Bladder 100 phase 3 trial [12, 13].

The PMS population was older than that of the avelumab + BSC arm of JAVELIN Bladder 100, as shown by the lower proportion aged ≤ 64 years (16.6% vs. 36.9%) and higher proportion aged ≥ 75 years (39.7% vs. 24.3%) [13, 26]. Furthermore, a small proportion of patients received prior PBC with the cisplatin‐containing (dd)MVAC regimen (2.0%), which was not permitted in the JAVELIN Bladder 100 trial [12, 13]. The PMS population compared with that of JAVELIN Bladder 100 included a lower proportion of patients whose response to prior PBC was CR (10.4% vs. 25.7%), with a correspondingly higher proportion whose response was PR (53.4% vs. 46.6%) or SD (32.9% vs. 27.7%) [24], further illustrating the differences between the study populations.

In the PMS population, baseline characteristics were generally balanced across subgroups, although some imbalances were observed that may have affected outcomes. The subgroup aged ≥ 75 years, compared with younger subgroups, included a lower proportion with an ECOG PS of 0, a higher proportion with renal impairment, and a higher proportion who had received prior carboplatin + gemcitabine, consistent with increased rates of comorbidity or frailty in older patients. Similarly, the subgroup who had received prior carboplatin + gemcitabine included a higher proportion of patients aged ≥ 75 years or with renal impairment than the prior cisplatin + gemcitabine subgroup. Compared with the subgroup who had a CR with prior PBC, subgroups who had prior PR and SD included higher proportions of patients with renal impairment and a higher frequency of prior carboplatin + gemcitabine treatment.

The primary objective of PMS was to assess safety. Despite the heterogeneous nature of the PMS population, the observed safety profile of avelumab maintenance mirrored findings from the JAVELIN Bladder 100 trial [5, 12, 13]. Although some numerical differences were observed, patterns of prespecified ADRs were comparable across all subgroups examined, and no new safety concerns were identified. Rates of any‐grade and grade ≥ 3 prespecified ADRs appeared lower in patients aged ≤ 64 years compared with those seen in the overall population [5], but were relatively consistent across other subgroups. The most common prespecified ADRs in all subgroups were infusion reaction and thyroid dysfunction, consistent with observations in the overall population. Patients aged ≤ 64 years or those who received cisplatin + gemcitabine as prior PBC appeared to have higher rates of hepatic function disorders compared with other subgroups. No apparent differences were observed among subgroups in the use of corticosteroids to manage ADRs.

The effectiveness of avelumab maintenance was observed across subgroups defined by age, prior PBC regimen, or BOR to prior PBC. Median TTF from the start of avelumab maintenance ranged from 4.4 to 5.2 months. In previous reports, median TTF was 4.6 months in the overall population and was also 4.6 months in a smaller real‐world study performed in Japan (J‐AVENUE; *N* = 79) [4, 5]. TTF provides an overall measure of treatment duration in clinical practice and is informative when assessing real‐world treatment patterns. However, TTF has limitations as an endpoint because it counts all reasons for discontinuation as events, including those unrelated to treatment safety or effectiveness (e.g., surgery, patient preference, or transfer to another hospital), which affects the interpretation of TTF data. All patients were observed for up to 52 weeks, and median OS was not reached in any subgroup; 12‐month OS rates in patients aged ≤ 64, 65 to 74, or ≥ 75 years were 77.6%, 82.7%, and 72.6%, respectively. Older age has been associated with not receiving systemic treatment in patients with aUC, which may be due to age‐related comorbidities or poor performance status, patient preferences, concerns about treatment‐related toxicities, or other considerations [19, 20, 21, 22]. However, our findings suggest that in patients with curatively unresectable UC who have received PBC, avelumab maintenance provides clinical benefits across age subgroups, including older patients.

OS rates at 12 months in patients with prior cisplatin + gemcitabine or carboplatin + gemcitabine treatment were 79.6% and 73.8%, respectively. The 12‐month OS rate in patients with prior (dd)MVAC was 88.9%, but this observation should be interpreted with caution because of the small number of patients in the (dd)MVAC subgroup (*n* = 9). Avelumab maintenance therapy is recommended for cisplatin‐eligible or ‐ineligible patients with aUC whose disease has not progressed after 1L PBC [8, 9, 10, 11]. A previous meta‐analysis of clinical trials reported that 1L cisplatin‐based chemotherapy was associated with increased objective response and complete response rates versus 1L carboplatin‐based chemotherapy in patients with aUC [29]. However, cisplatin‐ineligible patients may have a higher frequency of comorbidities and may be less fit than cisplatin‐eligible patients; these differences may affect prognosis irrespective of treatment. Consequently, more recent real‐world analyses adjusted for baseline covariates did not find any difference in outcomes in patients who had received 1L cisplatin‐based versus carboplatin‐based treatment [30, 31]. Findings from the subgroup analyses reported here, in addition to previous analyses [13, 16, 17], suggest that avelumab maintenance may provide clinical benefits in patients without disease progression after receiving cisplatin‐ or carboplatin‐based chemotherapy.

In patients whose prior BOR to PBC was CR, PR, or SD, 12‐month OS rates were 89.4%, 76.3%, and 75.8%, respectively. In previous real‐world studies, CR has been associated with more favorable outcomes with avelumab maintenance [16, 17]. In addition, in a subgroup analysis of patients enrolled in the JAVELIN Bladder 100 trial following a CR with 1L PBC, long‐term efficacy benefits were obtained with avelumab maintenance + BSC versus BSC alone in terms of both OS (median, 39.8 vs. 28.5 months; HR, 0.72) and PFS (median, 9.5 vs. 5.1 months; HR, 0.58), respectively [24].

Across all subgroups, the most common reasons for avelumab discontinuation were disease progression (60.0%–76.8%) and prespecified ADRs (5.4%–13.3%). In patients who discontinued avelumab and received subsequent treatment, the most common next‐line regimens were PBC (with a substantial proportion receiving retreatment with their previous regimen) and enfortumab vedotin. Of note, in Japan, the approval and reimbursement authorization of enfortumab vedotin as a later‐line treatment occurred while this study was ongoing [11, 32]; therefore, not all patients had the same next‐line treatment options. At data cutoff, most patients either remained on avelumab or had received next‐line treatment (approximately 64%–78% across different subgroups).

The PMS data reported here have several limitations. Data were collected from case report forms, and source data verification was not conducted. The study design and methods of patient assessment in the PMS were different to those used in clinical trials; thus, any comparisons with JAVELIN Bladder 100 data are for descriptive purposes only. The PMS was a noninterventional, observational study with no control group and a limited observation period of 52 weeks; therefore, it was not possible to confirm that patient outcomes observed were due to avelumab treatment. The study was not designed to compare outcomes between subgroups, and the subgroup analyses reported were post hoc and descriptive in nature; thus, no formal statistical comparisons or subgroup interaction testing were performed. Lastly, outcomes may have been affected by imbalances in measured or unmeasured confounding variables; thus, results should be interpreted with caution.

## 5 Conclusions

Analyses from this large PMS population in Japan showed the safety and effectiveness of avelumab maintenance therapy in heterogeneous subgroups defined by age, prior PBC regimen, or BOR to prior PBC. Findings reported here support previously reported analyses from the JAVELIN Bladder 100 phase 3 trial, demonstrating the favorable benefit–risk profile of avelumab maintenance therapy in patients with curatively unresectable UC in clinical practice. Overall, results suggest that avelumab maintenance is a suitable treatment for older and younger patients who have disease control (CR, PR, or SD) after receiving prior cisplatin‐ or carboplatin‐based chemotherapy.

## Author Contributions

**Masayoshi Nagata:** conceptualization, visualization, supervision, investigation, writing – review and editing. **Eiji Kikuchi:** conceptualization, visualization, supervision, investigation, writing – review and editing. **Taito Ito:** methodology, writing – review and editing. **Masashi Sato:** methodology, software, formal analysis, writing – review and editing. **Mie Ogi:** methodology, data curation, writing – review and editing. **Makiko Morita:** methodology, validation, writing – review and editing. **Masahiro Kajita:** conceptualization, visualization, writing – original draft, writing – review and editing. **Hiroyuki Nishiyama:** conceptualization, supervision, investigation, writing – review and editing.

## Ethics Statement

This study was conducted in accordance with Japanese regulations for Good Post‐Marketing Study Practice regulations (GPSP) [27]. GPSP regulations do not require the disclosure of all participating institutions. The study protocol was reviewed by all participating institutions and approval from ethics committees and institutional review boards was obtained based on the requirements of each institution. All patients provided written informed consent for publication.

## Conflicts of Interest

Masayoshi Nagata has provided speaker services for Astellas Pharma, Bayer Pharma Japan, Eisai Co., Ltd., Janssen Pharmaceutical K.K., Kyowa Kirin Co., Ltd., Merck Biopharma Co., Ltd., Tokyo, Japan, an affiliate of Merck KGaA, Darmstadt, Germany, and Sanofi K.K. Eiji Kikuchi has provided consulting or advisory services for Astellas Pharma, Bristol Myers Squibb, Chugai Pharmaceutical, Janssen, Kissei Pharmaceutical, Merck & Co., Kenilworth, NJ, and the healthcare business of Merck KGaA, Darmstadt, Germany; and has provided speaker services for Astellas Pharma, Bristol Myers Squibb, Chugai Pharmaceutical, Janssen, Kissei Pharmaceutical, Merck & Co., Kenilworth, NJ, Nippon Kayaku, the healthcare business of Merck KGaA, Darmstadt, Germany, and Taiho Pharmaceutical. Taito Ito, Masashi Sato, Mie Ogi, Makiko Morita, and Masahiro Kajita are employees of Merck Biopharma Co., Ltd., Tokyo, Japan, an affiliate of Merck KGaA, Darmstadt, Germany. Hiroyuki Nishiyama has provided speaker services for Astellas Pharma, Bristol Myers Squibb, Merck & Co., Kenilworth, NJ, Ono Pharmaceutical, and the healthcare business of Merck KGaA, Darmstadt, Germany, and has received research funding from Astellas Pharma and Chugai Pharmaceutical.

## Data Availability

Any requests for data by qualified scientific and medical researchers for legitimate research purposes will be subject to the healthcare business of Merck KGaA, Darmstadt, Germany's Data Sharing Policy. All requests should be submitted in writing to the healthcare business of Merck KGaA, Darmstadt, Germany's data sharing portal (https://www.emdgroup.com/en/research/our‐approach‐to‐research‐and‐development/healthcare/clinical‐trials/commitment‐responsible‐data‐sharing.html). When the healthcare business of Merck KGaA, Darmstadt, Germany, has a co‐research, co‐development, or co‐marketing or co‐promotion agreement, or when the product has been out‐licensed, the responsibility for disclosure might be dependent on the agreement between parties. Under these circumstances, the healthcare business of Merck KGaA, Darmstadt, Germany will endeavor to gain agreement to share data in response to requests.
